# A Step to Smart Fishways: An Autonomous Obstruction Detection System Using Hydraulic Modeling and Sensor Networks

**DOI:** 10.3390/s21206909

**Published:** 2021-10-18

**Authors:** Juan Francisco Fuentes-Pérez, Ana García-Vega, Francisco Javier Bravo-Córdoba, Francisco Javier Sanz-Ronda

**Affiliations:** 1 Department of Hydraulics and Hydrology, ETSIIAA, University of Valladolid, 34004 Palencia, Spain; jsanz@uva.es; 2 Centro Tecnológico Agrario y Agroalimentario Itagra.ct, 34004 Palencia, Spain; agvega@itagra.com (A.G.-V.); fjbravo@itagra.com (F.J.B.-C.)

**Keywords:** water-level sensors, low-cost, hydraulic modeling, fishways, neural networks, clogging

## Abstract

Stepped fishways are structures that allow the free movement of fish in transversal obstacles in rivers. However, the lack of or incorrect maintenance may deviate them from this objective. To handle this problem, this research work presents a novel low-cost sensor network that combines fishway hydraulics with neural networks programmed in Python (*Keras + TensorFlow*), generating the first autonomous obstruction/malfunction detection system for stepped fishways. The system is based on a network of custom-made ultrasonic water level nodes that transmit data and alarms remotely and in real-time. Its performance was assessed in a field study case as well as offline, considering the influence of the number of sensing nodes and obstruction dimensions. Results show that the proposed system can detect malfunctions and that allows monitoring of the hydraulic performance of the fishway. Consequently, it optimizes the timing of maintenance on fishways and, thus, has the potential of automatizing and reducing the cost of these operations as well as augmenting the service of these structures. Therefore, this novel tool is a step forward to achieve smart fishway management and to increase their operability.

## 1. Introduction

**Figure 1:**
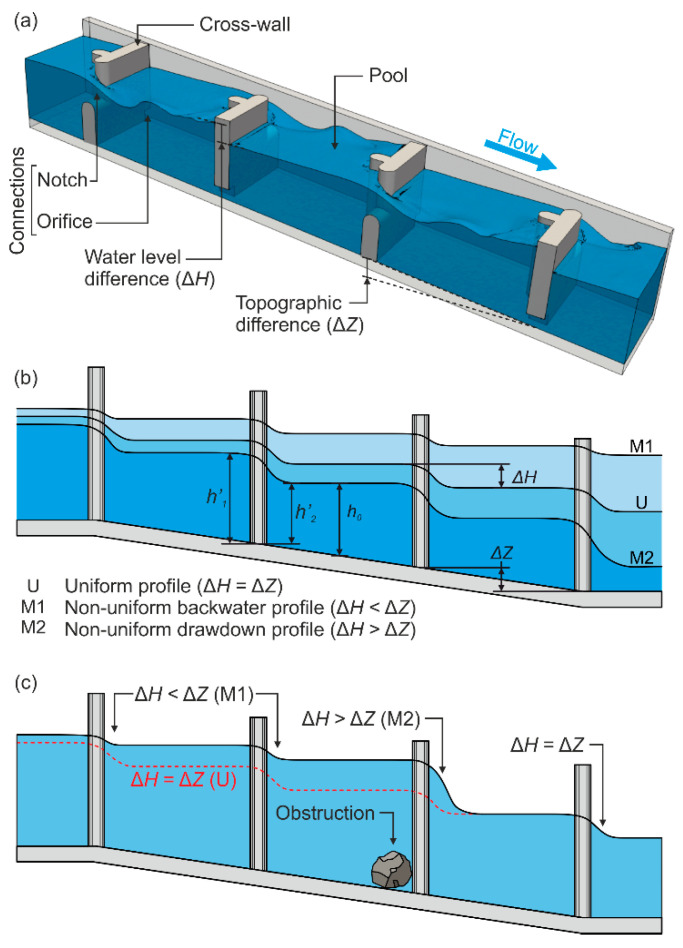
(**a**) A 3D sketch of a submerged notch and orifice stepped fishway. (**b**) Different scenarios in a fishway according to the natural variability of boundary conditions [15]. (**c**) Example of a non-uniform scenario forced by an obstruction.

Stepped fishways are the most extended solution to allow fish movement in transversal obstacles to rivers, such as weirs or dams [1,2]. These devices consist of a succession of cross-walls in a stepped pattern that divide the total height of an obstacle in a succession of pools with small drops between them (Figure 1). At the cross-walls, the water flows through connections (notches, slots, or submerged orifices), developing hydraulic conditions compatible with fish fauna capacities. Unfortunately, fishways are very sensitive structures due to the needed equilibrium between hydraulic and biological aspects inside them, and thus, their efficiency is often far from acceptable and, in some cases, even questioned [3,4,5]. This equilibrium can be disrupted by multiple circumstances such as an incorrect design, deviations during construction, the natural variability of rivers (which alter the boundary conditions upstream and downstream of the structure) [6], or incorrect maintenance [7]. Any situation altering their design–performance balance affects biological response inside them [8,9] and has the potential to deviate from their principal objective: allow the free movement of fish.

Recent serial studies on fishway performance assessment suggested that one of the most important sources of fishway malfunction is the absence of maintenance or its incorrect timing [7]. Stepped fishways are very sensitive to obstructions [9,10,11] and design guidelines suggest at least a weekly maintenance [12]. One of the main reasons for this need for frequent inspection is that connections between pools are usually of small width, enough to allow the fish passage, but, at the same time, minimizing the fishway discharge, which makes them susceptible to obstructions by drifting debris [12]. Although superficial trash racks or floating barriers at the water entrance of the fishway could diminish obstruction frequency, their use is not very common [7]. On the other hand, fishways are usually installed just in the river mainstem, and thus, they are in the first line of flood events, with the consequent impact of large woody debris and clogging. Therefore, to ensure the correct performance of fishways over time, it is necessary to implement inspection and maintenance plans [13,14]. However, maintenance is usually costly (as it entails periodical visits, sometimes to remote areas), requires a basic knowledge of fishway performance to be efficient, and, in some situations, it can be risky, as some fishways are located in difficult-to-access areas or present large dimensions. Consequently, maintenance is often dismissed and assumed as optional [7]. 

Considering the knowledge on fishway hydraulics [2,12,16] and the geometrical characteristics of a target fishway, it is possible to detect malfunctions just by observing the behavior of basic hydraulic parameters [6], for instance, water drops between pools. Any variation on the upstream and downstream boundary conditions of a fishway will generate a change from the ideal (uniform) design conditions to non-uniform scenarios inside it [1,16]. Non-uniformity consists of a variation of the design water levels inside a fishway from bottom-up to achieve a hydraulic equilibrium (Figure 1b). This also entails modification of the water drops, turbulence, volume of the pool, velocity, and flow patterns, as well as fish responses inside them [8,15,17]. In this sense, the presence of any obstruction will reduce the section of cross-wall connections (i.e., flow passage), increasing the water level in the pools above it (due to the new boundary condition for the upstream pools) and, consequently, generating a localized non-uniform scenario inside the fishway (Figure 1c). Therefore, considering this principle, it is possible to detect obstructions just by observing water level distributions in a fishway [14]. Likewise, obstructions directly reduce the fish passage area through connections (potentially affecting the discharge flowing through the fishway) and the increase in water drops is translated into a higher flow velocity which influences fish passage success [5].

Water level monitoring is a basic tool for many river applications. For instance, it is commonly used to permanently monitor river discharge or for flood forecasting [18,19,20,21,22]. There are multiple sensor alternatives to measure water levels in rivers (e.g., pressure sensor, floater, or ultrasonic), but the ones based on ultrasonic sensors are the most extended low-cost alternatives [20,22], as they do not require contact with water (and thus, lower sealing requirements and maintenance) and their sensing unit is relatively inexpensive [22]. Today, commercial ultrasonic water-level monitoring devices can be bought for less than 400€ (e.g., DL-MBX (www.decentlab.com, accessed on 15 August 2021), ELSYS ELT-2-HP (www.concept13.co.uk, accessed on 15 August 2021) and the material cost of custom-built units can be lower than 100€. 

Considering the above, the combination of low-cost sensing alternatives with basic hydraulic working principles of fishways could allow for the development of a smart sensor network able to generate maintenance alerts to optimize fishway management and performance. That is to say, triggering field visits only when needed and allowing less experienced people to detect malfunctions. To achieve this, the present research work develops a novel low-cost sensor network that combines fishway hydraulics with neural networks, generating the first autonomous obstruction/malfunction detection system for stepped fishways. Likewise, this work validates the system in a real fishway in a field test case and the network architecture is assessed in terms of the number of sensing nodes and obstacle dimensions. Results showed that the developed network was able to detect malfunctions and it allowed for the monitoring of the hydraulic performance of the fishway in real-time. In addition, the obtained results demonstrate that the system could be used for reinforcement learning of the developed algorithms, management purposes, and long-term hydraulic assessment of stepped fishways.

## 2. Materials and Methods

### 2.1. Fishway Hydraulic Modeling

The flow structure inside a stepped fishway is three-dimensional (3D) and varies according to the connections at the cross-walls. However, the water level distribution along the fishway pools (1D performance) can be easily calculated via an iterative bottom-up calculus considering (1) the boundary conditions upstream (discharge or water-level) and downstream (water-level), (2) discharge equations in the cross-wall-connections, and (3) the basic geometrical parameters of the fishway [1,15]. In this sense, the only unknown or variable parameters to solve the water-level distribution in each pool are the river water levels both upstream and downstream of the fishway. After the water level distribution is calculated, more complex variables can be estimated (such as the maximum velocity at the connections or the volumetric power dissipation in the pool).

To describe any of the working scenarios in a fishway (either uniform and non-uniform), it is possible to use two sets of equations [1]: set 1, Poleni’s equation [23] (Equation (1)) together with Villemonte’s submergence coefficient [24] (Equation (2)) to describe the performance of notches and slots, and set 2, the orifice equation derived from Torricelli’s law [25] (Equation (3)) together with a discharge coefficient [12,26,27,28] to describe the flow through an orifice. In each of the cross-walls, the total discharge can be expressed as the sum of the individual flows through the different connections.

(1) $$Q=\frac{2}{3}\cdot C_{s}\cdot b\cdot h_{1}^{1.5}\cdot \sqrt{2\cdot g}$$

(2) $$C_{s}=\beta_{0}{\left[1-{\left(\frac{h_{2}}{h_{1}}\right)}^{1.5}\right]}^{\beta_{1}}$$

(3) $$Q=C_{o}\cdot b_{o}\cdot a_{o}\cdot \sqrt{2\cdot g\cdot \Delta H}$$

where *g* stands for the gravity acceleration, *h*_1_ is the water level upstream the cross-wall (*h*_1_’) deducting the sill height (*p*), *h*_2_ is the water level upstream a cross-wall deducing *p*, *b* is the width of notches, slots, or orifices, *a* is the height of the orifice, *C* (*C_s_* and *C_o_*) stands for the discharge coefficients, and *β*_0_ and *β*_1_ are coefficients which depend on the geometry of the slot or notch and pool dimensions. Coefficient values for multiple fishway designs as well as the calculation procedure can be found in [1].

### 2.2. The Sensor Network

Considering that fishway hydraulic performance can be estimated only by observing water levels, our main objective was to develop a sensor network able to monitor this variable. The comparison of monitored water levels with the theoretical water level distribution in the fishway could give the necessary clues to identify anomalous performances related to obstructions. 

Although there are multiple alternatives to measure the water levels, those based on ultrasonic sensors have multiple advantages [22]. This type of sensor does not need to interact directly with the water, which reduces the sealing requirements and its maintenance. Likewise, contrary to other alternatives such as pressure sensor probes, ultrasonic sensors are installed outside the water, which allows for achieving more compact designs integrating the sensors, wireless communications, and removable energy sources like solar into a single unit. 

In addition, considering the large number of nodes of the network, i.e., sensors to be deployed (a maximum of one sensor per pool), the higher cost of commercial sensors (≤400€), and the disadvantages of closed-source technologies (e.g., they do not allow the custom programming), we decided to develop a new low-cost and market-ready ultrasound-based node, the MS Ultra (https://www.gea-ecohidraulica.org/GEA_en/sensors.php, accessed on 20 August 2021). 

The desired basic design characteristics for the new device were:

1. It needs to be able to precisely measure water levels (at least centimeter accuracy).
2. It has to be able to wirelessly transmit the information to a central gateway.
3. It has to be able to work in conjunction with other sensors autonomously.
4. It needs to be solar powered for a long time persistence.
5. It has to be low-cost technology (<130€, considering material and assembly costs).

Considering these basic characteristics, the sensor network architecture is described in the following sections.

#### 2.2.1. Hardware

The network is composed of (1) multiple water level monitoring nodes (MS Ultra) installed over the different pools of a fishway and (2) a central gateway, which communicates with the nodes individually, runs the algorithms, collects the data, triggers the alarms, and transmits the information to an online server. An alternative use case of a similar network architecture can be found in [29].

**Figure 2:**
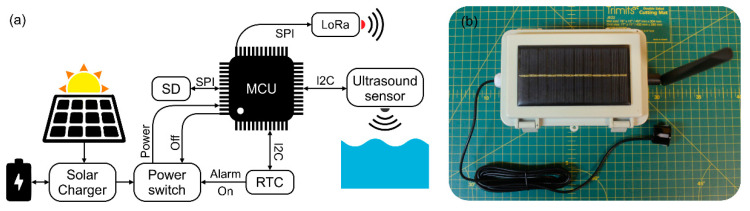
The architecture of the node. (**a**) Connections. (**b**) MS Ultra.

The nodes were designed to be used beyond this paper, thus, besides covering the listed characteristics, they were designed with other useful features to allow other potential uses (such as standalone monitoring). Their principal component is an Atmel ATSAMD21G18 microcontroller (Arduino compatible [30]). This microcontroller is connected to (1) a Long Range (LoRa) RFM95 radio module and a secure digital (SD) card via serial peripheral interface (SPI) connection, and (2) to a real-time clock (RTC, DS3231) and an ultrasonic sensor (JSN-SR04T) via an inter-integrated circuit (I2C) connection (Figure 2a). Likewise, an electronic power switch (Pololu 2808) makes it possible to control the MS Ultra’s ON/OFF cycles using RTC alarms. All components are soldered to a printed circuit board protected in a watertight (IP68) acrylonitrile butadiene styrene (ABS) housing (150 × 100 × 70 mm). The system is powered by an 18,650 Li-Ion battery, which is charged by a solar panel (6V, 1W) attached to the ABS box (Figure 2b).

The gateway consists of a small single-board computer (Raspberry Pi 4). This computer is connected to an Atmel ATSAMD21G18 microcontroller with an integrated LoRa RFM95 radio module and a 4G modem via serial connection. The computer is powered by a 30 A regulator connected to a 12 V battery and a solar panel of 80 W.

#### 2.2.2. Software

**Figure 3:**
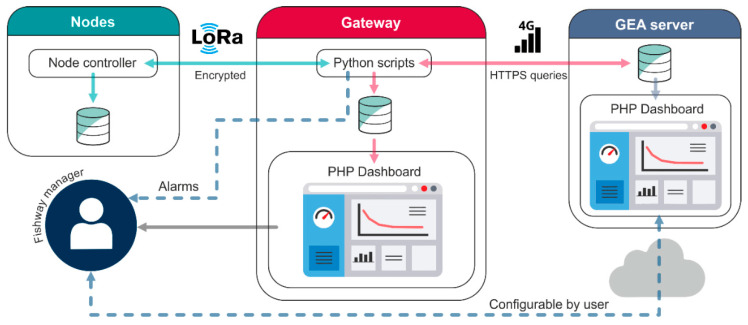
The general architecture of the sensor network.

The node (MS Ultra) and the microcontroller connected to the gateway are programmed in C using the Arduino integrated development environment (IDE) [30]. The single board computer is running the Raspberry Pi operating system (OS) with scheduled Python scripts (Figure 3). The code allows an encrypted communication between nodes and the gateway. Each node has its own address, which allows the gateway to identify each one and synchronize their transmission cycles (i.e., the gateway asks for data to each address).

In the programmed workflow, the gateway asks for data to a particular node, the node collects 15 distance measurements, discards possible invalid samples, computes the median, saves the data in the SD card, and transmits the information to the gateway. If the transmission is successful, the gateway returns a new wake-up time to the node, which configures a new wake-up alarm in the node and switches it off. This procedure allows for (1) saving power in the nodes by switching them off, (2) synchronizing the data transmission, and (3) controlling the sampling rate by the gateway. If a particular node is unresponsive, the gateway recursively tries to communicate with it during a programmable number of cycles (2 by default). After, if the communication is still not successful, it triggers a management alarm and continues with the rest of the nodes (Figure 3).

Once the gateway has collected a sample for each node, it runs an obstruction detection algorithm (depending on the active number of nodes) and triggers an obstruction alarm if this exists. Finally, data is saved in a local database (MySQL) as well as an online server (HTTPS queries + MySQL), for long time assessment (Figure 3). Further details on the online dashboard architecture and workflows can be found in [29].

### 2.3. Obstruction Detection Algorithm

Any deviation from the theoretical or simulated hydraulic performance of the fishway will indicate a possible obstruction event. A straightforward algorithm could check for the magnitude of any deviation to identify an obstruction or other malfunctions. However, in some cases, information related to geometrical peculiarities of the fishway could be missing, that is to say, specific geometrical deviations produced in the construction phase that differ from the original design project. Therefore, in this research work, we adopted a machine learning approach. Such approaches can learn how to detect obstructions without explicit programming of hydraulic rules, capturing any geometrical peculiarities as well as making possible reinforcement learning using observed real scenarios. 

Considering this, to classify anomalies into possible obstruction events, a multiclass classification artificial neural network was developed. First, different obstruction events were simulated, considering the geometry of the target fishway (submerged notch and orifice fishway; see Section 2.4. Study Site and Experimental Setup) and using the hydraulic model described above (see Section 2.1. Fishways Hydraulic Modeling). The physical result of an obstruction is a reduction of a flowing section in the connections of a cross-wall; thus, they were simulated by modifying the dimension of orifices (*a* and *b* in Equation (2)) in the equations and the sill of the notches (*p,* that directly affects to the notch area). In total, 42 scenarios were simulated, considering different levels of obstructions in cross-walls (7 obstruction scenarios for each cross-wall (only one obstructed cross-wall in each scenario) and 7 scenarios without obstruction) (Table 1). Due to the expected natural small water level oscillations on the pools (pulsed flow) and not being simulated by the equations, a normally distributed random noise (µ = 0 and α = 0.05 m) was added to the 7 simulated scenarios without obstruction. This could slightly reduce the detection capability of small obstructions but would make the algorithm more robust to false ones. Likewise, obstructions lower than 5 cm usually do not compromise the working performance of the fishway and they could be momentary (if permanent, they will aggravate with time). For all simulations, uniform performance was assumed as natural (without obstruction) behavior with a discharge of 0.250 m^3^/s and a mean water level on pools of 1.2 m (*h*_0_ = 1.20 m, *h*_1_’ = 1.33 m, *h*_2_’ = 1.08 m). Then, these simulations were randomly split into training (80%) and validation (20%) datasets. The final performance of the neural network was evaluated using an independent test of 12 real field scenarios (see Section 2.4).

The neural network was designed in Python (version 3.7) using *Keras* library (version 2.4.3). *Keras* (www.keras.io, 22 July 2021) is a free open-source Python library for developing and evaluating deep learning models making use of the numerical computation library *TensorFlow* (version 2.5) (www.tensorflow.org, 22 July 2021). The model consisted of a sequential network trained by the *Adam* optimizer (learning rate = 0.01 and number of interactions = 2000). The neural network structure was defined by a systematic search, testing different numbers of layers and neurons and computing their performance [31]. The final structure consisted of an input layer of 5 neurons (corresponding to the number of ultrasound sensors or sensing nodes), a hidden layer of 20 neurons activated by a sigmoid function, and an output layer of 6 neurons (corresponding to 5 possible obstruction events (obstruction in cross-wall 1 to 5) and a no obstruction event) activated by the *softmax* function to obtain the likelihood of each event. Once the design and training of the multiclass classification network were finished, the model could be exported to the gateway by saving its structure in JSON (JavaScript Object Notation) format and computed weight in HDF5 (Hierarchical Data Format version 5) format. 

### 2.4. Study Site and Experimental Setup

**Figure 4:**
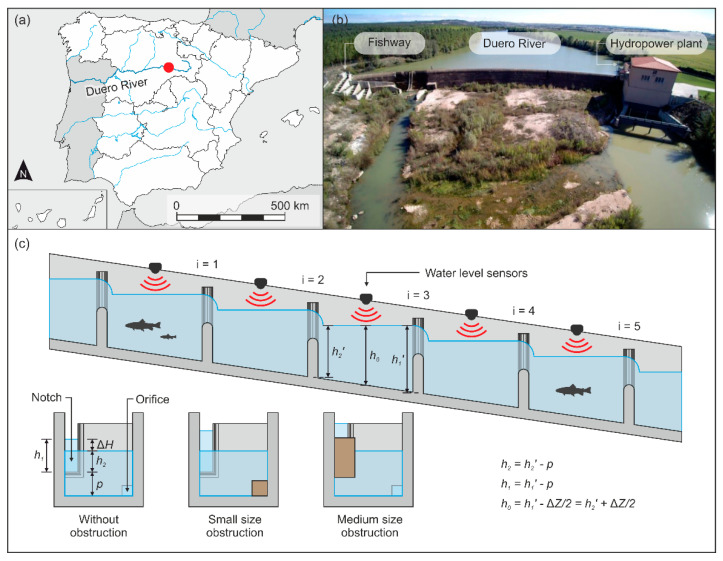
Study case. (**a**,**b**) Location. (**c**) Experimental setup.

The final performance of the proposed sensor network architecture and algorithms was field-tested in a fishway located in the Duero River, near Guma village in the northwest part of Spain (41°38′13.9″ N, 3°32′36.9″ W) (Figure 4a,b). The fishway is composed of 36 cross-walls with submerged notches and bottom orifices (notch width (*b_n_*) = 0.3 m; sill height (*p*) = 0.8 m; orifice size = 0.175 m (*b_o_*) × 0.175 m (*a_o_*)) and 35 pools (length = 2.6 m; width = 1.6 m; slope = 8.6%), with mean water drops (Δ*H*) of 0.25 m, mean water depth in the pools (*h*_0_) of 1.2 m, and volumetric power dissipation of 121 ± 10 W/m^3^ [32]. For testing and evaluation purposes, the sensor network was installed in the five uppermost pools (Figure 4c). All sensors were installed at the middle section of the pools to measure approximate *h*_0_, under the assumption of a more or less horizontal water level over the pool.

In each cross-wall (Figure 4c, from *i* = 1 to *i* = 5), two obstruction events were tested (10 tests in total + 2 scenarios without obstructions (beginning and end of tests)): (1) an small size obstruction, covering entirely the orifice with a wood board, and (2) a medium size obstruction, covering the lower part of the notch (20 cm) with a wood board but avoiding the water from spilling over the cross-wall. Only one cross-wall was obstructed in each test. All data were collected autonomously by the sensor network and stored in the gateway, as well as in the online server (Section 2.3).

### 2.5. Data Treatment and Validation

The analyses of collected data and the evaluation of the overall performances were performed using Python and Matlab R2019a.

To test that the hydraulic modeling was able to predict obstruction events, first field scenarios were graphically compared with simulated results, calculating the overall errors as well as the goodness of the fit between the model output and the measured values (*R*^2^, coefficient of determination). Then, the performance of the neural network algorithm was assessed using confusion matrices, for training, validation, and test datasets. 

In addition, the effect of the number of sensors and the possible effect of obstruction size in the algorithms was evaluated offline. For this, new artificial neural networks were developed using simulated data for training and validation, field data for testing, and reducing the number of input neurons (three sensing nodes: in the uppermost, middle, and down-most cross-walls; and two sensing nodes: in uppermost and down-most cross-walls). The final performance was assessed using confusion matrices and the evolution of classification error.

## 3. Results and Discussion

### 3.1. Test Datasets and Expected Hydraulic Performance

The hydraulic performance of fishways is a well-studied research subject [2,16,33,34,35,36] (among others); however, few research papers successfully apply new findings for its optimization [6,37]. A specific example is the non-uniform behavior of fishways [1,16]. Non-uniformity is a broadly studied phenomenon in fishways as a consequence of variations in river discharge and water levels. Despite the fact that this phenomenon directly modifies the hydraulic equilibrium inside the fishways, and therefore fish responses [8], it is rarely considered to improve or even analyze their performance. In this work, equations able to simulate non-uniform behavior are used to detect malfunctions in the performance of fishways and to develop an obstruction detection system with the potential of reducing maintenance costs as well as to optimize their operation by triggering alarms when maintenance is necessary. 

**Figure 5:**
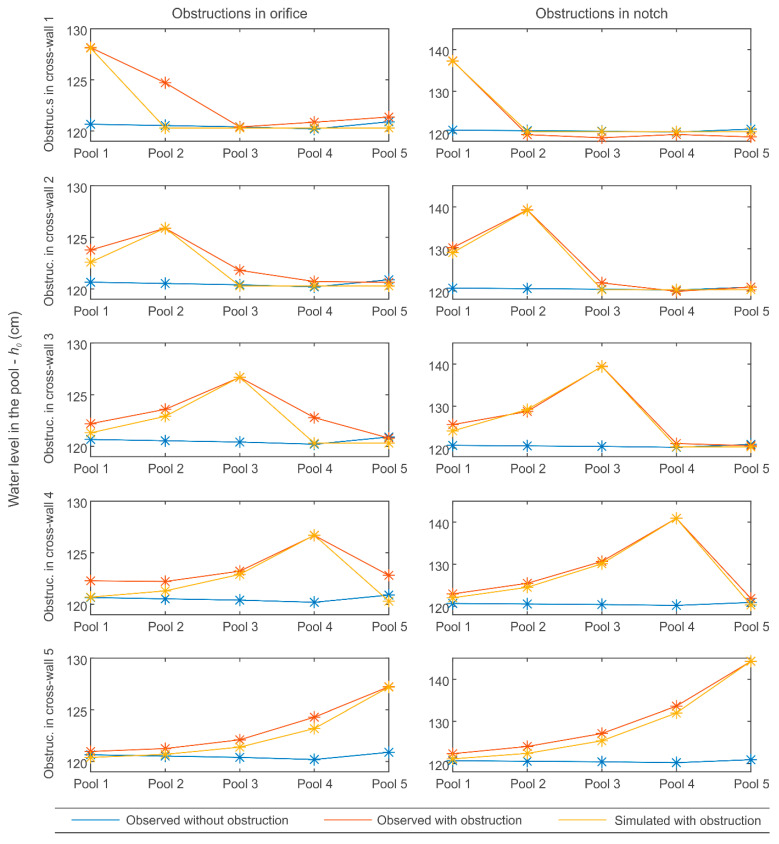
Scenarios tested in the field against 1D simulations of tested setups.

Considering the tested scenarios in the field, the sets of equations described in Section 2.1 are able to model the effect of obstructions in fishways (Figure 5). The goodness of the fit between model outputs and measured values is high (*R*^2^ = 0.974) and the mean absolute error is only 0.9 cm. The accuracy in the detection of obstructions at notches outperforms those of the orifices. For the latter, the equations predict a recovery in the water depth downstream of the obstructed orifice to the normal working conditions (≈1.2 m) but the measured values are significantly higher, with a more gradual recovery. This can be due to the three-dimensional performance of a fishway, where the flow over the connections in a cross-wall (in the case under study, orifice and notch) not only depends on their geometrical characteristics but also on the flow structure in the upstream pool. That is to say, an obstruction in an orifice modifies the flow structure in the pool below it, which in turn also modifies the discharge coefficients in the cross-wall connections below it. The magnitude of this modification will depend on the geometrical properties of the fishways (e.g., the arrangement between cross-wall connections, volume, and dimensions of the pool, deflectors, etc.). Even so, the predicted results are promising and they seem to be able to detect the presence of an obstruction. 

Regarding the expected results in other stepped fishway typologies, in [17], the same set of equations were used to simulate the non-uniform performance of a vertical slot fishway, obtaining a deviation of around 2% for the different studied water level profiles. Likewise, in a case study of depth prediction with the same set of equations in a submerged notch and bottom orifice fishway [6], a deviation of around 1.7% was obtained. Both error magnitudes are in agreement with the observed results, reinforcing the use of 1D modeling equations for uniform and non-uniform scenario prediction and, therefore, to feed the obstruction detection algorithm. 

### 3.2. Training, Validation, and Test of the Artificial Neural Network

Since the proposed hydraulic equations are able to predict the anomalies produced by an obstruction in a fishway, the observed differences from an expected performance (i.e., without obstruction) can directly provide a clue to its identification. Considering this, it is possible to use expected predictions to develop an algorithm to classify the likelihoods of different obstruction scenarios. To achieve this, in the present paper, a multiclass classification neural network has been developed, using a simulated dataset of 42 scenarios to train and validate the network and a field study case of 12 scenarios to test its final performance. 

**Figure 6:**
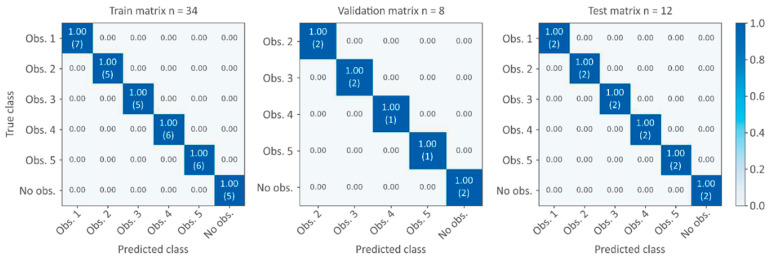
Confusion matrix for training, validation, and test datasets (42 simulated scenarios; 12 measured scenarios) using five nodes, i.e., a node per pool (obs. = obstruction; n = number of scenarios). The numbers in the matrix correspond to percentages (sum by row = 1.00) while the numbers between parentheses correspond to the number of scenarios.

Figure 6 shows the confusion matrix of training, validation, and test datasets. These matrices allow for visualizing the performance of the algorithm by representing in each row the instance of the observed class (true class) while representing in each column the instance of the predicted class. That is to say, in each row, it is possible to observe the percentage of classification by classes as well as to detect to which class corresponds the misclassifications.

When a node is installed in each pool, the performance of the neural network is perfect, that is to say, the network is able to accurately predict all the tested obstructions in the field.

Artificial neural networks have been previously used as an alternative to hydraulic simulations for real-time decision-making, modeling, forecasting, or failure-risk assessment [38]. They can provide fast responses for complex hydraulic behavior and solve hydraulic problems using just empirical data, leaving aside possible deterministic resolutions and integrating possible nonlinear relationships. In this sense, the proposed algorithm could be trained in the field for a specific fishway despite its complexity, by testing different obstructions on-site and using these datasets for training; that is to say, encapsulating its working principle in an artificial neural network [39]. Moreover, the initial network could be trained with simulated data and then reinforced with real data collected over time.

### 3.3. Effects of Number of Nodes

The number of nodes (i.e., number of sensors or measured water levels) will have a direct impact on the prediction accuracy and class probabilities. It was expected that the lower the number of nodes, the more difficult detection of small obstructions in not monitored cross-walls and the more probable misclassification between adjacent cross-walls. 

**Figure 7:**
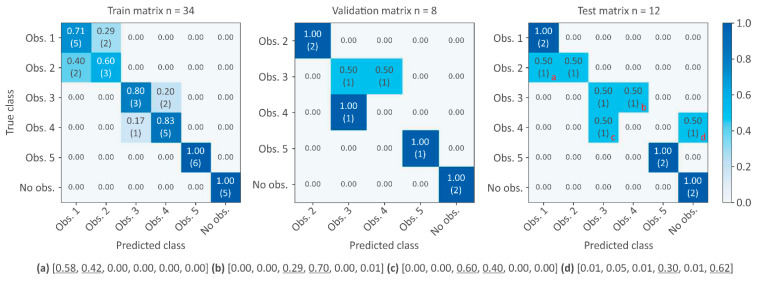
Confusion matrix for training, validation, and test datasets using three nodes. Misclassifications in the test dataset correspond to obstruction in the second cross-wall notch (**a**), obstruction in third cross-wall orifice (**b**), obstruction in the fourth cross-wall notch (**c**), and obstruction in the fourth cross-wall orifice (**d**) (class probabilities of misclassifications are indicated at the bottom of the picture).

**Figure 8:**
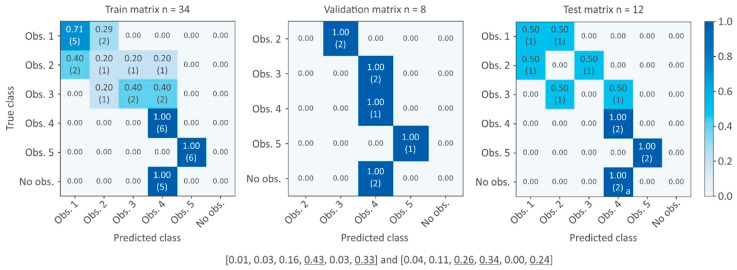
Confusion matrix for training, validation, and test datasets using two nodes. Misclassifications of no obstruction class in test dataset (class probabilities of misclassifications are indicated at the bottom of the picture).

Figure 7 and Figure 8 show the effect of reducing the number of nodes (three and two nodes, respectively) to predict the six possible classes after re-training the neural network. A setup with three nodes still performs correctly (Figure 7); the misclassifications are concentrated in adjacent cross-walls and the system would still be successful in triggering obstruction alarms. It is worth mentioning that in all misclassified classes, the second most probable class was the correct one (Figure 7). Therefore, the algorithm seems to perform well under this setup and the associated uncertainty due to the lack of information in the non-measured pools could be handled by providing, together with the obstruction class, the likelihood of all different classes. The tested obstruction types (notches vs. orifices) and sizes (Table 1) seem to not have a significant effect. Misclassifications seem to be related to the fact that without all the information in the non-monitored pools, multiple scenarios could provide the same water-level distribution. 

The class estimation is more challenging, with only a two-node setup (Figure 8). It is an extreme case of the previously tested node setups, where the lack of information in the non-monitored pools leads to a larger uncertainty, as multiple obstruction events could produce similar water level distributions. Considering the training dataset and the unbalance between obstruction (*n* = 35) and no obstruction scenarios (*n* = 7), in the test dataset, all no obstruction scenarios are classified as probable obstructions by the algorithm. However, no obstruction probability remains high, and therefore, false alarms could be avoided by establishing probability thresholds. 

### 3.4. Summary, Limitations, and Future Work

Results show that the best setup for the sensor network consisted of one node per pool as it allowed for the perfect detection of obstructions. However, the number of nodes directly affects the cost of the system and, thus, a setup with one sensor every two pools could be preferable when the main objective is the generation of alarms. The minimum requirements of the system are to detect obstructions (independently of cross-wall classification) and avoid misclassifications on no obstruction events; thus, the two-node setup could be of interest if a probability threshold is established. In this sense, considering that obstructions are more probable in the upstream cross-walls of the fishway (water intake and entrance of drifting debris), the installation of the system could be limited to this area. Additionally, during the development of this work, an interesting design alternative was raised: nodes with double ultrasonic sensors. With this, one node could register the water depth of two consecutive pools. The architecture of MS Ultra is compatible with this new design concept and it would reduce by half the number of necessary nodes while achieving the full fishway monitoring and thus, the highest accuracy at a lower cost. 

Regarding the training procedure, slight differences were observed between simulations and field scenarios, more noticeable in orifice obstructions. Thus, the use of real field data to train the artificial neural network could improve the reported classifications. In the same way, simulations are unable to fully collect the geometrical peculiarities of a fishway, which reinforces the use of onsite data to train the artificial neural network. Moreover, the training of the neural network can be periodically updated through scheduled scripts in the gateway using new data collected to improve its performance over time.

An additional possible improvement for the described system would be to detect multiple obstructions, although obstructions are expected to occur one by one and the system would still identify the anomaly (even if they are wrongly classified). This could be achieved by increasing the number of simulated scenarios, including multiple obstructions, or developing an artificial neural network for different sections of the fishway. In the same way, non-uniform natural scenarios are possible in the field, and thus the inclusion of obstruction events together with non-uniform performances would be of interest. Other malfunction events could be easily incorporated into the systems, such as abrupt water level reductions that could indicate maintenance or poaching. In addition, it could be interesting to test the performance of alternative classification schemes (e.g., a binary classifier for obstruction/no obstruction followed by a multiclass classifier only when required) as well as more advanced network models (e.g., 1D Convolutional Neural Network).

Regarding hardware, it is worth mentioning that following the same goal and working principle, different sensors could be used to measure the water levels (e.g., pressure sensor, lidar, floater, etc.) as well as fully alternative architectures such as cameras and artificial vision (possibly at a higher monetary and energy cost). Whichever the case, the selected system is going to be subject to conditions (different fishway dimensions or exposure) that must be assessed before its installation. For instance, in remote areas, mobile communication might not be accessible and thus another alternative would need to be considered (e.g., iridium satellite communication). In the same way, some fishways are directly exposed to flood events and, despite nodes being inside an IP68 graded enclosures, special attention should be taken to protect them from impacts. Thus, adaptability, scalability, and costs have been the main factors driving the proposed alternative. 

The developed system is just a step forwards to improve the operation of fishways, to achieve better management, and to make possible the optimization of their performance. The continuous monitoring of their hydraulic performance in conjunction with biological monitoring systems (e.g., radio/PIT/acoustic-tags or fish counters) could improve their understanding (which is still a pending task in the ecohydraulics research community [3,40]). This will make it possible to detect optimal working scenarios (understood as scenarios that trigger a higher number of fish passing through the fishway) and, in the end, to develop management strategies and technologies to autonomously achieve these optimal scenarios (e.g., automatic sluice gates). For instance, the system could be directly used for real-time fishway discharge gauging (mandatory in some European countries [41]), detecting flow increments related to fish migration peaks [41,42], and optimizing the detection of fishway by fish (increasing auxiliary discharge) or the entrance of fish into the fishway (increasing or reducing the drop with automatic sluice gates). 

## Figures and Tables

**Table 1:** Geometrical modifications in each of the cross-walls to simulate different obstructions events.

Orifice Obstructions ^1^				Notch Obstructions ^2^				
New *a_o_* (m)	0.10	0.05	0.00	New *p* (m)	0.90	1.00	1.10	1.20
New *b_o_* (m)	0.10	0.05	0.00					

^1^ Orifice original value: *a* = 0.175 m and *b* = 0.175 m. ^2^ Notch sill original value: *p* = 0.8 m. In Equation (1): *h*_1_ = *h*_1_’ − *p*.

## Data Availability

Data are available upon reasonable request to the corresponding author.
